# Adiposity and Metabolic Indices in the Diagnosis and Histological Stage Association of Metabolic Dysfunction-Associated Steatotic Liver Disease

**DOI:** 10.3390/jcm14238365

**Published:** 2025-11-25

**Authors:** Lorena del Rocio Ibarra-Reynoso, Nemry Rodriguez-Hernandez, Maria-Luisa Lazo-de-la-Vega-Monroy, Juana Rosalba Garcia-Ramirez, Yeniley Ruiz-Noa, Benjamin Jordan-Perez, Serafin Garnelo-Cabañas, Veronica Muñoz-Cornejo, Monica del Carmen Preciado-Puga

**Affiliations:** 1 Department of Medical Sciences, Health Sciences Division, Leon Campus, University of Guanajuato, León de los Aldama 37670, Gto, Mexico; lorena.ibarra@ugto.mx (L.d.R.I.-R.); n.rodriguezhernandez@ugto.mx (N.R.-H.); mlazo@ugto.mx (M.-L.L.-d.-l.-V.-M.); yeni.rn@hotmail.com (Y.R.-N.); 2 Department of Medicine and Nutrition, Health Sciences Division, Leon Campus, University of Guanajuato, León de los Aldama 37670, Gto, Mexico; rosy_gr@hotmail.com (J.R.G.-R.); veromunoz9418@gmail.com (V.M.-C.); 3 Department of Surgery, General Hospital Leon, León de los Aldama 37670, Gto, Mexico; benjajordan1@gmail.com (B.J.-P.); seragarnelo@hotmail.com (S.G.-C.)

**Keywords:** metabolic dysfunction-associated steatotic liver disease, adiposity indices, metabolic dysfunction-associated steatohepatitis

## Abstract

**Background:** Metabolic dysfunction-associated steatotic liver disease (MALSD) is defined as the excessive accumulation of triglycerides in the liver in the presence of at least one cardiometabolic risk factor and liver biopsy remains the diagnostic gold standard. This study aimed to evaluate the diagnostic performance of adiposity and metabolism related indices for the non-invasive detection of MASLD and the metabolic dysfunction-associated steatohepatitis (MASH). **Methods:** A cross-sectional study was conducted in 161 Mexican adults undergoing laparoscopic cholecystectomy, during which liver biopsies were obtained for histological evaluation. Indices such as the Hepatic Steatosis Index (HSI), the Triglyceride–Glucose index (TyG), TyG-BMI (TyG adjusted for body mass index), and TyG-WC (TyG adjusted for waist circumference), among others, were calculated. **Results:** Of the 161 participants, 66 were diagnosed with MASLD, and 50 of them had histological evidence of MASH. All adiposity and metabolic indices evaluated were significantly higher in MASLD patients compared with controls. Logistic regression identified HSI, TyG, TyG-BMI, and TyG-WC as independently associated with MASLD and MASH, with TyG showing the strongest association. Correlation analyses demonstrated that TyG-BMI and TyG-WC were most strongly associated with histological features of MASH. Receiver operating characteristic curve analyses showed that TyG-WC had the highest diagnostic accuracy for MASLD (AUC 0.721, 95% CI 0.641–0.802) and MASH (AUC 0.735, 95% CI 0.648–0.823), while TyG-BMI displayed high sensitivity (0.758 for MASLD; 0.780 for MASH). **Conclusions:** Triglyceride–glucose-based indices, particularly TyG-WC and TyG-BMI, showed the highest diagnostic performance for detecting MASLD and MASH, suggesting that these indices may serve as practical, non-invasive tools for identifying individuals at risk.

## 1. Introduction

The term non-alcoholic fatty liver disease (NAFLD) has traditionally been used to describe a spectrum of conditions characterized by the presence of macrovesicular steatosis in more than 5% of hepatocytes, in the absence of other specific etiologies [1]. Recently, an international consensus proposed a change in terminology, replacing NAFLD with metabolic dysfunction-associated steatotic liver disease (MASLD), in order to better reflect its pathophysiological basis [2].

MASLD is defined as the excessive accumulation of triglycerides in the liver in the presence of at least one cardiometabolic risk factor [2]. The term encompasses a wide spectrum of conditions, including simple steatosis (metabolic dysfunction-associated steatotic liver, MASL), metabolic dysfunction-associated steatohepatitis (MASH), as well as progressive stages such as fibrosis and cirrhosis [2].

MASLD is currently the most prevalent liver disease worldwide and has become the second leading indication for liver transplantation in the United States, surpassed only by alcohol-related liver disease [3]. Its prevalence is rising in parallel with the global obesity epidemic, with an estimated worldwide prevalence of approximately 25% in the general population [4]. This prevalence, however, varies according to factors such as race/ethnicity, geographic region, clinical setting, and the diagnostic method employed [1].

Metabolic dysfunction-associated steatotic liver disease (MASLD) carries a well-recognized risk of progression from isolated steatosis to more advanced stages, including metabolic dysfunction-associated steatohepatitis (MASH), fibrosis, cirrhosis, and ultimately hepatocellular carcinoma. Disease progression is largely driven by the burden of metabolic dysfunction, with type 2 diabetes mellitus, obesity, and hypertension representing the strongest risk factors. It is estimated that nearly one-third of individuals with MASLD develop lipotoxicity, hepatocellular injury, and lobular inflammation—the defining features of MASH—which in turn accelerate fibrogenesis and increase the likelihood of adverse liver-related outcomes [5].

Liver biopsy remains the gold standard for the diagnosis of MASLD and for staging disease progression toward MASH and advanced fibrosis [6]. The histopathological hallmarks of MASLD include: (1) hepatocellular triglyceride accumulation; (2) centrilobular hepatocellular injury, which is most pronounced in the acinar zone; (3) cytoskeletal disruption manifested as hepatocellular ballooning with or without Mallory–Denk bodies; (4) parenchymal inflammation, predominantly composed of lymphocytes and macrophages, with occasional neutrophilic infiltration; and (5) perisinusoidal fibrosis characterized by collagen deposition in the space of Disse. These features collectively define the transition from simple steatosis to MASH and are essential for histological diagnosis and disease staging [7].

Although liver biopsy remains the gold standard for diagnosing MASLD and staging its progression to MASH, its use is limited by several drawbacks, including invasiveness, high cost, sampling variability, and the risk of complication. These limitations have fueled the search for reliable, non-invasive alternatives capable of capturing the metabolic and structural determinants of the disease [8].

Given the limitations of liver biopsy, several adiposity and metabolic indices have been proposed as non-invasive alternatives to evaluate the risk of MASLD and its progression. Indices such as Visceral Adiposity Index (VAI), Body Adiposity Index (BAI), Body Roundness Index (BRI), Relative Fat Mass (RFM), Abdominal Volume Index (AVI), and Weight-Adjusted Waist Index (WWI) estimate body fat and its distribution, while Hepatic Steatosis Index (HSI), Triglyceride–glucose index (TyG), TyG adjusted for Body Mass Index (TyG-BMI), and TyG adjusted for Waist Circumference (TyG-WC) incorporate metabolic parameters linked to insulin resistance and hepatic steatosis [9,10,11,12]. These tools are practical, low-cost, and accessible, making them attractive for early identification of MASLD and advanced forms like MASH, despite this, the validation of multiple adiposity and TyG-derived indices against liver biopsy has not been widely explored.

The primary objective of this study was to evaluate the diagnostic performance of adiposity and metabolic indices including VAI, BAI, BRI, RFM, AVI, WWI, HSI, TyG, TyG-BMI and TyG-WC for the non-invasive detection of MASLD and its advanced stages, such as MASH, using liver biopsy as the reference standard. In the present study we hypothesize that the indices are useful for the detection of MASLD and MASH

## 2. Materials and Methods

### 2.1. Participants

A cross-sectional study was conducted in the central region of Mexico, enrolling 161 individuals aged 18 to 60 years who underwent laparoscopic cholecystectomy. During the procedure, liver biopsy samples were collected for histological evaluation.

### 2.2. Eligibility Criteria

#### 2.2.1. Inclusion Criteria

- No Reported significant alcohol consumption (≤20 g/day for women and ≤30 g/day for men) in the 12 months preceding enrollment.
- Had no prior diagnosis of liver disease, including alcoholic liver disease, viral or autoimmune hepatitis, cirrhosis, hepatocellular carcinoma, or primary sclerosing cholangitis.
- Had no secondary hepatic steatosis due to medications (e.g., glucocorticoids, methotrexate, amiodarone, tamoxifen, certain antivirals), rapid or severe weight loss, metabolic disorders, or inflammatory bowel disease.
- Had no evidence of choledocholithiasis before, during, or after surgery.

#### 2.2.2. Exclusion Criteria

- Participants were excluded if they had:
- Rapid or severe weight loss within the last 6 months or recent bariatric surgery.
- Pregnancy or breastfeeding.
- Severe systemic diseases affecting metabolic status (e.g., uncontrolled type 1 diabetes, severe thyroid disorders).
- Conditions preventing safe liver biopsy (e.g., coagulopathy, thrombocytopenia, or active infection).

### 2.3. Clinical and Metabolic Assessment

Fasting blood samples were obtained to measure glucose, triglycerides, high-density lipoprotein (HDL), and low-density lipoprotein (LDL) cholesterol levels which were determined by standard colorimetric methods with a chemistry analyzer (Auto KEM II, Kontrollab, Rome, Italy). Blood pressure, weight, and height were measured using standardized procedures. Waist circumference was measured at the midpoint between the last rib and the iliac crest, while hip circumference was measured at the level of the pubic symphysis. Body mass index (BMI) was calculated as weight in kilograms divided by height in meters squared. Dyslipidemia was defined as the presence of hypertriglyceridemia (triglycerides ≥ 150 mg/dL or use of lipid-lowering therapy) or low HDL cholesterol (≤40 mg/dL in men, ≤50 mg/dL in women, or use of lipid-lowering therapy) [2]. Metabolic syndrome (MS) was defined according to the current harmonization criteria, as the presence of any three of five cardiometabolic risk factors [13].

### 2.4. Adiposity Indices

Anthropometric and metabolic indices were calculated using established formulas, including VAI, BAI, BRI, AVI, RFM, WWI, HSI, TyG, TyG-BMI and TyG-WC (Table 1).

### 2.5. Histological Analysis

Histopathological diagnosis was performed using the Kleiner scoring system and the NAFLD Activity Score (NAS) by an experienced pathologist at HGL. MASLD was diagnosed in patients with histologically confirmed hepatic steatosis together with at least one cardiometabolic criterion: BMI ≥ 25 kg/m^2^ or elevated waist circumference (>94 cm in men, >80 cm in women); impaired glucose metabolism (fasting glucose ≥ 100 mg/dL, 2 h glucose ≥ 140 mg/dL, HbA1c ≥ 5.7%, type 2 diabetes, or antidiabetic treatment); elevated blood pressure (≥130/85 mmHg or antihypertensive therapy); hypertriglyceridemia (≥150 mg/dL or lipid-lowering therapy); or reduced HDL-cholesterol (≤40 mg/dL in men, ≤50 mg/dL in women or lipid-lowering therapy) [2]. For advanced stages of the disease, patients presenting with at least 1 point in each component of the Kleiner scoring system (steatosis, lobular inflammation, and hepatocellular ballooning) were classified as having metabolic dysfunction-associated steatohepatitis (MASH). Patients diagnosed with MASLD who did not meet criteria for MASH were classified as MASL (metabolic dysfunction–associated steatosis of the liver).

### 2.6. Statistical Analysis

Normality of continuous variables was assessed using the Kolmogorov–Smirnov test. For comparisons between groups, Student’s *t*-test or the Mann–Whitney U test was used, depending on data distribution. Logistic regression analyses were performed with adiposity and metabolic indices as independent variables to evaluate their association with MASLD and MASH diagnoses. The Bonferroni correction was applied. Spearman correlation coefficients were calculated to explore the relationship between indices and histological features of MASH. Additionally, the diagnostic performance of each index was assessed by calculating the area under the receiver operating characteristic curve (AUC), 95% confidence intervals (CI), optimal thresholds (Youden index), positive predictive value (PPV), negative predictive value (NPV), sensitivity, and specificity. Statistical analyses were conducted using SPSS version 25 (IBM SPSS Statistics, New York, NY, USA) and GraphPad Prism 10.4.0 (GraphPad Software, Inc. en Boston, MA, USA), with a two-sided *p*-value < 0.05 considered statistically significant. A post hoc power analysis was performed for the main indices TyG-BMI and TyG-WC, yielding Cohen’s d values of 0.62 and 0.77, respectively, with corresponding statistical powers of 97% and 99.8%, indicating that the sample size was sufficient to detect significant differences.

### 2.7. Ethical Considerations

This study was conducted in accordance with the principles of the Declaration of Helsinki [21] and the Mexican General Health Law on Health Research [22], which classifies it as involving more than minimal risk. The research protocol was reviewed and approved by Research and Ethics Committees of the General Hospital León, Secretary of Health of the state of Guanajuato on 13 January 2017 and the Ethics Committee of the University of Guanajuato (Approval No. CIBIUG-P03-2017) on 5 September 2017. Written informed consent was obtained from all participants prior to enrollment.

## 3. Results

### 3.1. General Characteristics of the Study Groups

A total of 161 participants were included in this study, of whom 95 were controls and 66 were diagnosed with MASLD. Among the MASLD cohort, histological analysis identified MASH in 50 patients. Participant characteristics and key clinical and metabolic differences are summarized in Table 2. Obesity and metabolic syndrome were significantly more frequent in MASLD patients than in controls (57.6% vs. 29.5% and 59.1% vs. 31.6%, respectively; *p* = 0.001 for both), while diabetes and dyslipidemia showed no significant differences between groups. Group comparisons revealed no significant sex differences between MASLD and control groups (*p* = 0.472). However, patients with MASLD were notably older (*p* = 0.001). Anthropometric measurements were significantly elevated in the MASLD cohort, including weight, BMI, waist circumference, and hip circumference (*p* < 0.05 for all parameters). All adiposity indices under investigation—including VAI, BAI, BRI, AVI, WWI, RFM, HSI, TyG, TyG-BMI and TyG-WC—were significantly higher in MASLD patients relative to controls (all *p* ≤ 0.05). Additionally, MASLD subjects demonstrated elevated systolic blood pressure (*p* = 0.006), fasting glucose (*p* = 0.034), and liver function markers: GGT, AST, and ALT (all *p* < 0.01). The MASLD group also had higher total cholesterol, VLDL, and triglyceride levels (*p* < 0.01), while HDL and LDL levels did not differ significantly.

### 3.2. Correlation Between Adiposity Indices and Histological Features of MASH

Spearman correlation analyses were performed to evaluate the relationship between adiposity indices and the histological features of MASH, including steatosis, lobular inflammation, and hepatocellular ballooning (Table 3). Indices AVI, TyG-BMI, and TyG-WC showed the highest correlations with steatosis (r = 0.304, 0.276, 0.294, and 0.352, respectively; all *p* < 0.001). Additionally, TyG-BMI and TyG-WC showed the strongest correlations with both inflammation (*r* = 0.267, *p* < 0.001; *r* = 0.222, *p* = 0.005) and ballooning (*r* = 0.230, *p* = 0.003; *r* = 0.242, *p* = 0.002.).

### 3.3. Logistic Regression Analysis

We performed logistic regression analyses to evaluate the association of adiposity indices with MASLD and MASH (Table 4). Multivariate models were adjusted for age and sex. Several indices, including BAI, BRI, AVI, HSI, TyG, TyG-BMI and TyG-WC were independently associated with both MASLD and MASH. Among them, TyG showed the strongest association with MASLD and MASH.

### 3.4. Diagnostic Performance of Adiposity and Metabolic Indices for MASLD and MASH

**Figure 1:**
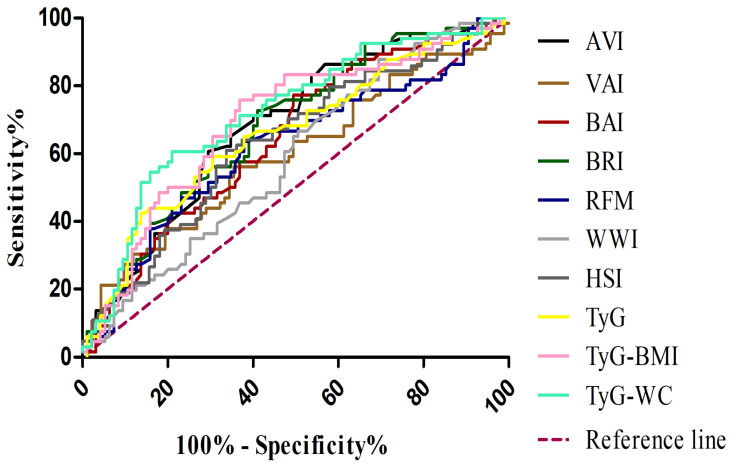
Receiver operating characteristic (ROC) curves for adiposity and metabolic indices in the diagnosis of MASLD. VAI, visceral adiposity index (brown line); BAI, body adiposity index (red line); BRI, body roundness index (dark green line); AVI, abdominal volume index (black line); WWI, weight-adjusted waist index (light grey line); RFM, relative fat mass (blue line); HSI, hepatic steatosis index (dark grey line); TyG, triglyceride–glucose index (yellow line); TyG-BMI, TyG adjusted for body mass index (pink line); TyG-WC, TyG adjusted for waist circumference (light green line); MASLD, metabolic dysfunction–associated steatotic liver disease.

Receiver Operating Characteristic (ROC) curve analyses were performed to evaluate the diagnostic performance of all indices for MASLD compared with control subjects (Table 5) (Figure 1). Among the indices tested, TyG-WC demonstrated the highest diagnostic accuracy, with an AUC of 0.721 (95% CI: 0.641–0.802), a sensitivity of 0.606, and a specificity of 0.789. TyG-BMI also showed good performance, with an AUC of 0.694 (95% CI: 0.610–0.778), a sensitivity of 0.758, and a specificity of 0.632.

**Figure 2:**
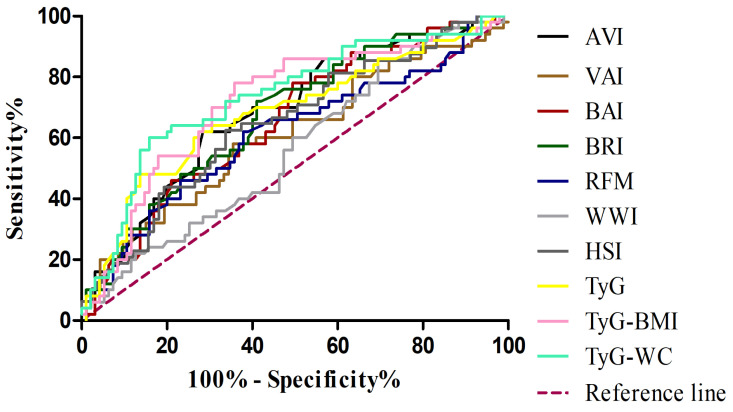
Receiver operating characteristic (ROC) curves for adiposity and metabolic indices in the diagnosis of MASH. VAI, visceral adiposity index (brown line); BAI, body adiposity index(red line); BRI, body roundness index(dark green line); AVI, abdominal volume index(black line); WWI, weight-adjusted waist index (light grey line); RFM, relative fat mass(blue line); HSI, hepatic steatosis index(dark grey line); TyG, triglyceride–glucose index(yellow line); TyG-BMI, TyG adjusted for body mass index (pink line); TyG-WC, TyG adjusted for waist circumference(light green line); MASH, metabolic dysfunction–associated steatohepatitis.

**Figure 3:**
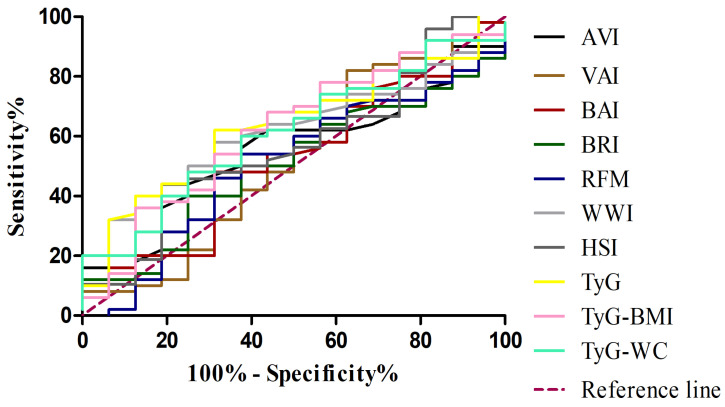
Receiver operating characteristic (ROC) curves for adiposity and metabolic indices in distinguishing MASH from MASL. VAI, visceral adiposity index (brown line); BAI, body adiposity index (red line); BRI, body roundness index (dark green line); AVI, abdominal volume index (black line); WWI, weight-adjusted waist index (light grey line); RFM, relative fat mass (blue line); HSI, hepatic steatosis index(dark grey line); TyG, triglyceride–glucose index (yellow line); TyG-BMI, TyG adjusted for body mass index (pink line); TyG-WC, TyG adjusted for waist circumference (light green line); MASH, metabolic dysfunction–associated steatohepatitis; MASL, metabolic dysfunction–associated steatosis of the liver.

In the subsequent analysis, comparing MASH patients with controls (Table 6) (Figure 2), a similar pattern was observed, although some metabolic indices showed improved diagnostic performance. In this setting, the TyG-WC index demonstrated the highest discriminative ability (AUC 0.735; 95% CI: 0.648–0.823), with particularly high specificity (0.842), while the TyG-BMI also performed well (AUC 0.717; 95% CI: 0.627–0.806), showing the highest sensitivity (0.780). When evaluating the ability of adiposity- and metabolism-related indices to discriminate MASH from MASL patients (Table 7) (Figure 3), most markers showed limited diagnostic accuracy. Classical adiposity indices, including VAI, BAI, BRI, WWI, and RFM, displayed AUC values close to 0.50, indicating poor discriminatory capacity. Similarly, HSI and AVI achieved only modest performance. By contrast, indices incorporating triglyceride–glucose parameters showed relatively better results. The TyG index reached the highest AUC (0.634; 95% CI: 0.489–0.778), with balanced sensitivity (0.62) and specificity (0.69). Both TyG-BMI and TyG-WC also exceeded an AUC of 0.60, maintaining moderate discriminative capacity.

## 4. Discussion

The prevalence of MASLD has risen markedly over the past decades, largely driven by the global epidemics of obesity and metabolic dysfunction [3]. Within this spectrum, the progression to MASH, characterized by hepatic inflammation and hepatocellular injury with or without fibrosis, represents a critical stage, as it is strongly associated with an increased risk of cirrhosis and hepatocellular carcinoma [23]. Consequently, there is a growing need for reliable, non-invasive markers capable of distinguishing patients at risk of progression from those with simple steatosis, in order to optimize early detection, risk stratification, and timely intervention [8].

Consistent with previous reports, patients with MASLD in our cohort exhibited a clinical and biochemical profile characterized by higher body weight, body mass index (BMI), anthropometric measures such as waist and hip circumference, systolic blood pressure, triglycerides, total cholesterol, as well as elevated liver enzymes (AST and ALT). These alterations underscore the well-established association between MASLD and cardiometabolic risk factors, including obesity, metabolic syndrome, hypertension, and dyslipidemia [24,25,26].

In line with our findings, all evaluated adiposity indices including VAI, BAI, BRI, AVI, WWI, RFM, HSI, TyG, TyG-BMI, and TyG-WC were significantly higher in patients with MASLD compared to controls.

Anthropometric and metabolic indices serve as surrogate markers of central obesity and insulin resistance, and when integrated with metabolic parameters, they provide a more accurate reflection of the systemic metabolic burden associated with MASLD [10].

Although results revealed that the TyG index had the highest odds ratio, suggesting a strong independent association with MASLD and MASH, the ROC curve analysis demonstrated that TyG-BMI and TyG-WC achieved the largest areas under the curve. While TyG alone is a robust predictor of risk, the combined indices provide superior discriminatory power in distinguishing MASLD and MASH patients from controls. These findings suggest that integrating TyG with anthropometric measures enhances diagnostic accuracy by capturing both metabolic dysfunction and adiposity-related components of disease pathophysiology. The TyG index, calculated from fasting plasma glucose and triglyceride levels, is a widely used surrogate marker for insulin resistance. Modified parameters derived from TyG, such as TyG-WC and TyG-BMI, have demonstrated enhanced performance in identifying individuals with IR, reflecting both adiposity and metabolic dysfunction more accurately [27]. Previous studies have shown that TyG-BMI exhibits a stronger correlation with the degree of hepatic steatosis than TyG alone, suggesting that higher TyG-BMI values are associated with more severe liver fat accumulation [28]. This emphasizes the added value of incorporating anthropometric measures into metabolic indices to better reflect hepatic lipid burden. A previous study reported that TyG-WC demonstrated the strongest performance in predicting hepatic steatosis among MASLD patients diagnosed through abdominal ultrasound examination, highlighting the added diagnostic value of integrating waist circumference with metabolic parameters [10].

Consistent with these findings, in our study TyG-BMI and TyG-WC exhibited the strongest correlations with key histological features of MASH, including steatosis, lobular inflammation, and hepatocellular ballooning. Previous studies have reported a significant increasing trend in the risk of developing MASH, significant fibrosis, advanced fibrosis, and cirrhosis as TyG-BMI values rise, highlighting its potential as a predictor of disease progression [29,30].

The performance of all indices declined when discriminating MASH patients from those with MASL. This suggests that although these indices effectively reflect the metabolic derangements and adiposity burden associated with advanced disease, they may lack sufficient sensitivity to the histological hallmarks, such as inflammation, ballooning, and fibrosis, that differentiate MASH from simple steatosis.

The hepatic steatosis index (HSI) has been widely used as a non-invasive screening tool for hepatic steatosis [20]. However, in our biopsy-confirmed cohort its performance was modest, showing only limited ability to discriminate MASLD and MASH from controls. These findings are in line with previous reports where HSI exhibited weak to moderate correlations with liver fat and failed to adequately distinguish between different degrees of steatosis severity [31]. Building on this, it is important to note that HSI was originally developed to identify hepatic fat content rather than histological features such as inflammation or ballooning. This limitation may explain its modest performance in distinguishing MASLD and MASH. In contrast, indices that incorporate both adiposity and metabolic parameters, such as TyG-BMI and TyG-WC, demonstrated superior diagnostic value in our study, particularly in capturing the systemic and histological complexity of advanced disease.

Insulin resistance is a key driver of MASLD progression. Elevated TyG-derived indices reflect early defects in insulin signaling, promoting increased lipolysis and free fatty acid delivery to the liver [32]. The resulting metabolic overload enhances de novo lipogenesis and impairs β-oxidation, leading to hepatic lipid accumulation and lipotoxicity [33]. These lipid intermediates are associated with activation of stress and inflammatory pathways such as JNK and NF-κB, which have been observed in hepatocellular injury and more advanced histological stages of MASLD, including MASH. [34].

As a limitation in the present study is the design cross-sectional, which limits causal inference. Another limitation is that all participants were recruited among patients undergoing cholecystectomy for gallstone disease. Since gallstone disease is a common condition in both the MASLD and control groups, this shared characteristic may have influenced the results and limits the generalizability of our findings to the broader population. Therefore, future studies including other populations should be conducted.

Our study has notable strengths. The use of biopsy-proven MASLD and MASH provides robust validation of the associations between adiposity indices and histological features, which is rarely achieved in noninvasive studies. Furthermore, the simultaneous evaluation of multiple adiposity and TyG-derived indices allowed us to identify those with the greatest diagnostic potential, offering a comparative perspective that enhances clinical relevance.

In conclusion in this study, triglyceride–glucose-based indices, particularly TyG-WC and TyG-BMI, consistently demonstrated superior diagnostic performance for the detection of MASLD and MASH compared with classical adiposity indices, therefore de AUCs were moderate. These findings should be interpreted with caution; however, they could support in the future the use of the TyG-derived indices as practical, non-invasive tools for identifying individuals at risk of MASLD in clinical settings, which could help prioritize patients for further evaluation or intervention. Future studies should validate these findings in larger and more diverse cohorts and explore their utility in longitudinal monitoring and prediction of disease progression, considering other factors like potential confounders such as dietary intake, physical activity, or medication.

## Figures and Tables

**Table 1:** Adiposity and metabolic indices used in the study with corresponding formulas.

Abbreviation	Index name	Formula
VAI	Visceral Adiposity Index [14]	Men: VAI = [WC/(39.68 + 1.88 × BMI)] × (TG/1.03) × (1.31/HDL-C) Women: VAI = [WC/(36.58 + 1.89 × BMI)] × (TG/0.81) × (1.52/HDL-C)
BRI	Body Roundness Index [15]	BRI = 364.2 − 365.5 × √[1 − (WC/(2π))^2^/(0.5 × height)^2^]
AVI	Abdominal Volume Index [16]	AVI = [2 × WC^2^ + 0.7 × (WC − HC)^2^]/1000
BAI	Body adiposity index [17]	BAI = (Hip (cm)/Height^1.5^ (m)) − 18
RFM	Relative fat mass [18]	RFM = 64 − (20 × Height (m)/WC (m)) + (12 × sex), sex = 0 (men), 1 (women)
WWI	Weight-Adjusted Waist Index [19]	WWI = WC (cm)/√Weight (kg)
HSI	Hepatic steatosis index [20]	HSI = 8 × (ALT/AST) + BMI + 2 (if diabetes) + 2 (if female)
TyG	Triglyceride–Glucose Index [10]	TyG = ln [TG × FPG/2]
TyG-BMI	TyG–Body Mass Index [10]	TyG-BMI = TyG × BMI
TyG-WC	TyG–Waist Circumference [10]	TyG-WC = TyG × WC

WC, waist circumference (cm); HC, hip circumference (cm); BMI, body mass index (kg/m^2^); TG, triglycerides (mmol/L); HDL-C, high-density lipoprotein cholesterol (mmol/L); FPG, fasting plasma glucose (mg/dL); ALT, alanine aminotransferase (U/L); AST, aspartate aminotransferase (U/L).

**Table 2:** Clinical and biochemical characteristics of control and MASLD groups.

Characteristics	Control	MASLD	*p* Value *
	*n* = 95	*n* = 66	
Sex, *n* (%)			
Female	83 (87.3)	55 (83.3)	0.472
Male	12 (12.7)	11 (16.7)	
Age (years, mean ± SD)	35.5 ± 12.11	42.11 ± 10.8	0.001
Weight (kg, mean ± SD)	69.5 ± 12.8	76.01 ± 13.4	0.002
Height (m, mean ± SD)	1.58 ± 0.06	1.57 ± 0.07	0.542
BMI (kg/m^2,^ mean ± SD)	27.8 ± 5.2	30.69 ± 5.2	0.001
Waist Circumference (cm, Median [25th, 75th Percentile])	99 (86–105)	104 (98–108.25)	<0.001
Hip circumference (cm, Median [25th, 75th Percentile])	100.2 (90–108)	107 (100.75–112)	0.001
Diabetes, *n* (%)	7 (7.4%)	10 (15.2%)	0.126
Obesity (BMI ≥ 30 kg/m^2^), *n* (%)	28 (29.5%)	38 (57.6%)	0.001
Dyslipidemia, *n* (%)	67 (70.5%)	49 (74.2%)	0.721
Metabolic syndrome, *n* (%)	30 (31.6%)	39 (59.1%)	0.001
VAI (Median [25th, 75th Percentile])	4.8 (3.34–7.7)	6.4 (3.9–11.07)	0.037
BAI (mean ± SD)	32.6 ± 7	36 ± 6.49	0.001
BRI (mean ± SD)	5.8 ± 1.8	6.81 ± 1.67	<0.001
AVI (mean ± SD)	18.9 ± 4.4	21.38 ± 3.89	<0.001
WWI (mean ± SD)	11.6 ± 0.89	11.89 ± 0.96	0.044
RFM (Median [25th, 75th Percentile])	43.4 (39.1–245.8)	45.47 (40.8–47.39)	0.010
HSI (mean ± SD)	38.45 ± 5.9	41.57 ± 6.4	0.003
TyG (mean ± SD)	8.55 ± 0.54	8.8 ± 0.61	0.001
TyG-BMI (mean ± SD)	239.49 ± 53.48	273.21 ± 54.78	<0.001
TyG-WC (mean ± SD)	826.94 ± 119.43	918.29 ± 118.06	<0.001
Systolic blood pressure (mmHg, Median [25th, 75th Percentile])	115 (110–124)	120.5 (112–134.25)	0.006
Diastolic blood pressure (mmHg, mean ± SD)	71.9 ± 10.2	73.08 ± 11.4	0.527
Glucose (mg/dL, Median [25th, 75th Percentile])	92.9 (82.3–98)	93 (87.7–108.5)	0.034
GGT (U/L, Median [25th, 75th Percentile])	19 (13.2–31.3)	31.5 (18.2–61.9)	<0.001
HDL (mg/dL, Median [25th, 75th Percentile])	42.9 (36.3–50.9)	44 (38–51.4)	0.542
CT (mg/dL, mean ± SD)	157.9 ± 29.6	171.7 ± 34.65	0.006
LDL (mg/dL, Median [25th, 75th Percentile])	86.6 (72.5–103.1)	91.1 (70.8–117)	0.260
VLDL (mg/dL, Median [25th, 75th Percentile])	22.6 (14.3–31.7)	28.06 (19.4–41.3)	0.011
TG (mg/dL, Median [25th, 75th Percentile])	109.5 (73–153)	145.3 (97.9–211)	0.003
AST (U/L, Median [25th, 75th Percentile])	20 (16.8–26)	24 (18.6–36.7)	0.007
ALT (U/L, Median [25th, 75th Percentile])	21 (15–31)	26.5 (19.6–40.7)	0.005

BMI, body mass index; VAI, visceral adiposity index; BAI, body adiposity index; BRI, body roundness index; AVI, abdominal volume index; WWI, weight-adjusted waist index; RFM, relative fat mass; HSI, hepatic steatosis index; TyG, triglyceride–glucose index; TyG-BMI, TyG adjusted for BMI; TyG-WC, TyG adjusted for WC; GGT, gamma-glutamyl transferase; HDL, high-density lipoprotein cholesterol; LDL, low-density lipoprotein cholesterol; VLDL, very-low-density lipoprotein cholesterol; TG, triglycerides; AST, aspartate aminotransferase; ALT, alanine aminotransferase. * *p*-values were calculated using Student’s *t*-test for normally distributed continuous variables, Mann–Whitney U test for non-normally distributed continuous variables, and Chi-square test for categorical variables.

**Table 3:** Correlation of Adiposity and Metabolic Indices with Histological Features of MASH.

Indices	Steatosis		Inflammation		Ballooning	
	Correlation Coefficient	*p* Value *	Correlation Coefficient	*p* Value *	Correlation Coefficient	*p* Value *
VAI	0.128	0.109	0.046	0.566	0.063	0.430
BAI	0.202	0.01	0.174	0.027	0.183	0.020
BRI	0.259	0.001	0.156	0.048	0.175	0.027
AVI	0.276	<0.001	0.184	0.019	0.211	0.007
WWI	0.128	0.104	−0.048	0.547	0.038	0.634
RFM	0.155	0.049	0.079	0.321	0.092	0.246
HSI	0.187	0.023	0.184	0.026	0.202	0.014
TyG	0.242	0.002	0.119	0.134	0.152	0.054
TyG-BMI	0.294	<0.001	0.267	<0.001	0.230	0.003
TyG-WC	0.352	<0.001	0.222	0.005	0.242	0.002

VAI, visceral adiposity index; BAI, body adiposity index; BRI, body roundness index; AVI, abdominal volume index; WWI, weight-adjusted waist index; RFM, relative fat mass; HSI, hepatic steatosis index; TyG, triglyceride–glucose index; TyG-BMI, TyG adjusted for body mass index; TyG-WC, TyG adjusted for waist circumference. * Correlation coefficients were calculated using Spearman’s rank correlation.

**Table 4:** Association of Adiposity and Metabolic Indices with MASLD and MASH.

Indices	MASLD				MASH			
	Univariate		Multivariate *		Univariate		Multivariate *	
	OR (95% CI)	*p* Value	OR (95% CI)	*p* Value	OR (95% CI)	*p* Value	OR (95% CI)	*p* Value
VAI	1.06 (0.992–1.134)	0.086	1.03 (0.97–1.10)	0.243	1.048 (0.983–1.117)	0.148	1.026 (0.963–1.093)	0.432
BAI	1.08 (1.028–1.133)	0.002	1.092 (1.034–1.15)	0.001	1.074 (1.021–1.129)	0.006	1.085 (1.025–1.149)	0.005
BRI	1.404(1.16–1.698)	<0.001	1.46 (1.19–1.80)	<0.001	1.337 (1.103–1.621)	0.003	1.402 (1.131–1.738)	0.002
AVI	1.156 (1.066–1.253)	<0.001	1.17 (1.07–1.27)	<0.001	1.137 (1.047–1.234)	0.002	1.162 (1.061–1.273)	0.001
WWI	1.435 (1.00–2.05)	0.05	1.46 (0.997–2.15)	0.052	1.249 (0.872–1.788)	0.225	1.236 (0.841–1.816)	0.281
RFM	1.052 (0.998–1.109)	0.058	1.19 (1.083–1.328)	<0.001	1.047 (0.989–1.108)	0.113	1.162 (1.044–1.294)	0.006
HSI	1.085 (1.026–1.147)	0.004	1.094 (1.031–1.161)	0.003	1.088 (1.026–1.153)	0.005	1.100 (1.033–1.172)	0.003
TyG	2.57 (1.45–4.56)	0.001	2.00 (1.081–3.718)	0.027	2.968 (1.612–5.463)	<0.001	2.28 (1.184–4.426)	0.014
TyG-BMI	1.012 (1.005–1.018)	<0.001	1.011 (1.004–1.018)	0.001	1.012 (1.005–1.019)	0.001	1.011 (1.004–1.018)	0.001
TyG-WC	1.007 (1.004–1.010)	<0.001	1.006 (1.003–1.009)	<0.001	1.006 (1.003–1.01)	<0.001	1.006 (1.003–1.009)	<0.001

MASLD, metabolic dysfunction-associated steatotic liver disease; MASH, metabolic dysfunction-associated steatohepatitis; VAI, visceral adiposity index; BAI, body adiposity index; BRI, body roundness index; AVI, abdominal volume index; WWI, weight-adjusted waist index; RFM, relative fat mass; HSI, hepatic steatosis index; TyG, triglyceride–glucose index; TyG-BMI, TyG adjusted for body mass index; TyG-WC, TyG adjusted for waist circumference; OR, odds ratio; CI, confidence interval. * Multivariate logistic regression models were adjusted for age and sex.

**Table 5:** Diagnostic Performance of Adiposity and Metabolic Indices for MASLD versus Control Subjects.

Indices	AUC	95% CI	Threshold	PPV	NPV	Sensitivity	Specificity
VAI	0.597	0.506–0.689	6.1406	0.523	0.678	0.561	0.645
BAI	0.649	0.564–0.734	32.3225	0.520	0.761	0.773	0.505
BRI	0.678	0.595–0.761	6.0439	0.551	0.756	0.727	0.589
AVI	0.683	0.60–0.765	20.4090	0.553	0.749	0.712	0.600
WWI	0.585	0.497–0.673	11.0520	0.467	0.783	0.879	0.305
RFM	0.619	0.529–0.709	44.1029	0.538	0.710	0.636	0.621
HSI	0.635	0.545–0.726	40.5720	0.556	0.709	0.609	0.663
TyG	0.657	0.570–0.774	9.0064	0.682	0.683	0.424	0.863
TyG-BMI	0.694	0.61–0.778	247.8707	0.588	0.789	0.758	0.632
TyG-WC	0.721	0.641–0.802	902.3981	0.666	0.742	0.606	0.789

VAI, visceral adiposity index; BAI, body adiposity index; BRI, body roundness index; AVI, abdominal volume index; WWI, weight-adjusted waist index; RFM, relative fat mass; HSI, hepatic steatosis index; TyG, triglyceride–glucose index; TyG-BMI, TyG adjusted for body mass index; TyG-WC, TyG adjusted for waist circumference; AUC, area under the receiver operating characteristic curve; CI, confidence interval; Threshold, optimal cutoff value determined by the Youden index; PPV, positive predictive value; NPV, negative predictive value.

**Table 6:** Diagnostic Performance of Adiposity and Metabolic Indices for MASH versus Control Subjects.

Indices	AUC	95% CI	Threshold	PPV	NPV	Sensitivity	Specificity
VAI	0.608	0.508–0.708	6.1406	0.462	0.744	0.580	0.645
BAI	0.655	0.563–0.746	32.3225	0.453	0.813	0.780	0.505
BRI	0.672	0.581–0.763	6.0439	0.480	0.799	0.720	0.589
AVI	0.683	0.593–0.774	21.4537	0.534	0.781	0.620	0.716
WWI	0.56	0.463–0.657	11.0520	0.394	0.805	0.860	0.305
RFM	0.614	0.514–0.713	44.1029	0.462	0.756	0.620	0.621
HSI	0.649	0.552–0.747	40.5720	0.493	0.770	0.625	0.663
TyG	0.681	0.586–0.776	8.8141	0.544	0.783	0.620	0.726
TyG-BMI	0.717	0.627–0.806	253.2597	0.534	0.847	0.780	0.642
TyG-WC	0.735	0.648–0.823	917.7715	0.66	0.799	0.600	0.842

VAI, visceral adiposity index; BAI, body adiposity index; BRI, body roundness index; AVI, abdominal volume index; WWI, weight-adjusted waist index; RFM, relative fat mass; HSI, hepatic steatosis index; TyG, triglyceride–glucose index; TyG-BMI, TyG adjusted for body mass index; TyG-WC, TyG adjusted for waist circumference; AUC, area under the receiver operating characteristic curve; CI, confidence interval; Threshold, optimal cutoff value determined by the Youden index; PPV, positive predictive value; NPV, negative predictive value.

**Table 7:** Diagnostic Performance of Adiposity and Metabolic Indices for MASH versus MASL Subjects.

Indices	AUC	95% CI	Threshold	PPV	NPV	Sensitivity	Specificity
VAI	0.524	0.348–0.699	3.8050	0.804	0.399	0.820	0.375
BAI	0.523	0.362–0.685	36.6627	0.827	0.296	0.480	0.688
BRI	0.492	0.340–0.644	8.9464	1.000	0.270	0.140	1.000
AVI	0.554	0.405–0.702	22.0546	0.846	0.299	0.440	0.750
WWI	0.386	0.244–0.529	13.166	1.000	0.257	0.080	1.000
RFM	0.489	0.328–0.650	48.9364	1.000	0.266	0.120	1.000
HSI	0.568	0.406–0.729	43.6658	0.879	0.315	0.438	0.813
TyG	0.634	0.489–0.778	8.8077	0.861	0.366	0.620	0.688
TyG-BMI	0.623	0.466–0.779	270.2166	0.838	0.344	0.620	0.625
TyG-WC	0.611	0.463–0.759	943.7845	0.857	0.315	0.480	0.750

VAI, visceral adiposity index; BAI, body adiposity index; BRI, body roundness index; AVI, abdominal volume index; WWI, weight-adjusted waist index; RFM, relative fat mass; HSI, hepatic steatosis index; TyG, triglyceride–glucose index; TyG-BMI, TyG adjusted for body mass index; TyG-WC, TyG adjusted for waist circumference; AUC, area under the receiver operating characteristic curve; CI, confidence interval; Threshold, optimal cutoff value determined by the Youden index; PPV, positive predictive value; NPV, negative predictive value.

## Data Availability

The data associated with the paper are available from the corresponding author on reasonable request.

## References

[1] Rinella M.E., Neuschwander-Tetri B.A., Siddiqui M.S., Abdelmalek M.F., Caldwell S., Barb D., Kleiner D.E., Loomba R. (2023). AASLD Practice Guidance on the clinical assessment and management of nonalcoholic fatty liver disease. Hepatology.

[2] Rinella M.E., Lazarus J.V., Ratziu V., Francque S.M., Sanyal A.J., Kanwal F., Romero D., Abdelmalek M.F., Anstee Q.M., Arab J.P. (2023). A multisociety Delphi consensus statement on new fatty liver disease nomenclature. J. Hepatol..

[3] Cotter T.G., Rinella M. (2020). Nonalcoholic Fatty Liver Disease 2020: The State of the Disease. Gastroenterology.

[4] Younossi Z.M. (2019). Non-alcoholic fatty liver disease—A global public health perspective. J. Hepatol..

[5] Devasia A.G., Ramasamy A., Leo C.H. (2025). Current Therapeutic Landscape for Metabolic Dysfunction-Associated Steatohepatitis. Int. J. Mol. Sci..

[6] Martinou E., Pericleous M., Stefanova I., Kaur V., Angelidi A.M. (2022). Diagnostic Modalities of Non-Alcoholic Fatty Liver Disease: From Biochemical Biomarkers to Multi-Omics Non-Invasive Approaches. Diagnostics.

[7] Younossi Z.M., Loomba R., Anstee Q.M., Rinella M.E., Bugianesi E., Marchesini G., Neuschwander-Tetri B.A., Serfaty L., Negro F., Caldwell S.H. (2018). Diagnostic modalities for nonalcoholic fatty liver disease, nonalcoholic steatohepatitis, and associated fibrosis. Hepatology.

[8] Tincopa M.A., Loomba R. (2024). Noninvasive Tests to Assess Fibrosis and Disease Severity in Metabolic Dysfunction-Associated Steatotic Liver Disease. Semin. Liver Dis..

[9] Zhou T., Ding X., Chen L., Huang Q., He L. (2025). Visceral adiposity index as a predictor of metabolic dysfunction-associated steatotic liver disease: A cross-sectional study. BMC Gastroenterol..

[10] Forouzesh P., Kheirouri S., Alizadeh M. (2025). Predicting hepatic steatosis degree in metabolic dysfunction-associated steatotic liver disease using obesity and lipid-related indices. Sci. Rep..

[11] van Kleef L.A., Michel M., Savas M., Pustjens J., van de Laar R., Koehler E., van Rossum E.F., Janssen H.L., Schattenberg J.M., Brouwer W.P. (2025). A Comparison of the Predictive Value of 12 Body Composition Markers for Metabolic Dysfunction-Associated Steatotic Liver Disease, At-Risk Metabolic Dysfunction-Associated Steatohepatitis, and Increased Liver Stiffness in a General Population Setting. Am. J. Gastroenterol..

[12] Wang J., He W., Cai X., Hu Z., Peng Y., Chen X., Yang P., Zeng X., Chen S., Wang D. (2025). Relative fat mass and risk of metabolic dysfunction associated steatotic liver disease and severe hepatic steatosis in U.S. adults: Analysis of NHANES 2017–2020 data. BMC Gastroenterol..

[13] Alberti K.G.M.M., Eckel R.H., Grundy S.M., Zimmet P.Z., Cleeman J.I., Donato K.A., Fruchart J.-C., James W.P.T., Loria C.M., Smith S.C. (2009). Harmonizing the metabolic syndrome: A joint interim statement of the International Diabetes Federation Task Force on Epidemiology and Prevention; National Heart, Lung, and Blood Institute; American Heart Association; World Heart Federation; International Atherosclerosis Society; and International Association for the Study of Obesity. Circulation.

[14] Jakubiak G.K., Badicu G., Surma S., Waluga-Kozłowska E., Chwalba A., Pawlas N. (2025). The Visceral Adiposity Index and its usefulness in the prediction of cardiometabolic disorders. Nutrients.

[15] Thomas D.M., Bredlau C., Bosy-Westphal A., Mueller M., Shen W., Gallagher D., Maeda Y., McDougall A., Peterson C.M., Ravussin E. (2013). Relationships between body roundness with body fat and visceral adipose tissue emerging from a new geometrical model. Obesity.

[16] Perona J.S., Schmidt-RioValle J., Fernández-Aparicio Á., Correa-Rodríguez M., Ramírez-Vélez R., González-Jiménez E. (2019). Waist Circumference and Abdominal Volume Index Can Predict Metabolic Syndrome in Adolescents, but only When the Criteria of the International Diabetes Federation are Employed for the Diagnosis. Nutrients.

[17] Bergman R.N., Stefanovski D., Buchanan T.A., Sumner A.E., Reynolds J.C., Sebring N.G., Xiang A.H., Watanabe R.M. (2011). A better index of body adiposity. Obesity.

[18] Woolcott O.O., Bergman R.N. (2018). Relative fat mass (RFM) as a new estimator of whole-body fat percentage—A cross-sectional study in American adult individuals. Sci. Rep..

[19] Park Y., Kim N.H., Kwon T.Y., Kim S.G. (2018). A novel adiposity index as an integrated predictor of cardiometabolic disease morbidity and mortality. Sci. Rep..

[20] Lee J.-H., Kim D., Kim H.J., Lee C.-H., Yang J.I., Kim W., Kim Y.J., Yoon J.-H., Cho S.-H., Sung M.-W. (2010). Hepatic steatosis index: A simple screening tool reflecting nonalcoholic fatty liver disease. Dig. Liver Dis..

[21] World Medical Association (2025). World Medical Association Declaration of Helsinki: Ethical Principles for Medical Research Involving Human Participants. JAMA.

[22] Secretaria de Salud (1987). Reglamento de la Ley General de Salud en Materia de Investigaciones Para la Salud. Ley Gen Salud [Internet]. http://www.diputados.gob.mx/LeyesBiblio/regley/Reg_LGS_MIS.pdf.

[23] European Association for the Study of the Liver (EASL), European Association for the Study of Diabetes (EASD), European Association for the Study of Obesity (EASO) (2024). EASL-EASD-EASO Clinical Practice Guidelines on the management of metabolic dysfunction-associated steatotic liver disease (MASLD). J. Hepatol..

[24] Kong L., Yang Y., Li H., Shan Y., Wang X., Shan X. (2023). Prevalence of nonalcoholic fatty liver disease and the related risk factors among healthy adults: A cross-sectional study in Chongqing, China. Front. Public Health.

[25] Lu Y., Hu L., Song J., Wan J., Chen H., Yin J. (2021). Gallstone disease and nonalcoholic fatty liver disease in patients with type 2 diabetes: A cross-sectional study. BMC Endocr. Disord..

[26] Byrne C.D., Armandi A., Pellegrinelli V., Vidal-Puig A., Bugianesi E. (2025). Μetabolic dysfunction-associated steatotic liver disease: A condition of heterogeneous metabolic risk factors, mechanisms and comorbidities requiring holistic treatment. Nat. Rev. Gastroenterol. Hepatol..

[27] Sánchez-García A., Rodríguez-Gutiérrez R., Mancillas-Adame L., González-Nava V., González-Colmenero A.D., Solis R.C., Álvarez-Villalobos N.A., González-González J.G. (2020). Diagnostic Accuracy of the Triglyceride and Glucose Index for Insulin Resistance: A Systematic Review. Int. J. Endocrinol..

[28] Wang M., Chang M., Shen P., Wei W., Li H., Shen G. (2023). Application value of triglyceride-glucose index and triglyceride-glucose body mass index in evaluating the degree of hepatic steatosis in non-alcoholic fatty liver disease. Lipids Health Dis..

[29] Zhang F., Han Y., Wu Y., Bao Z., Zheng G., Liu J., Li W. (2024). Association between triglyceride glucose-body mass index and the staging of non-alcoholic steatohepatitis and fibrosis in patients with non-alcoholic fatty liver disease. Ann. Med..

[30] Huang X., Hu Y., Dong L., Zhu L., Li J., Hu J., Shao W., Peng Y., Liu F. (2025). TyG-BMI as a superior predictor of MAFLD and pre-MAFLD in Chinese adults: A cross-sectional study. BMC Gastroenterol..

[31] Keating S.E., Parker H.M., Hickman I.J., Gomersall S.R., Wallen M.P., Coombes J.S., Macdonald G.A., George J., Johnson N.A. (2017). NAFLD in clinical practice: Can simple blood and anthropometric markers be used to detect change in liver fat measured by ^1^H-MRS?. Liver Int..

[32] Samuel V.T., Shulman G.I. (2012). Mechanisms for insulin resistance: Common threads and missing links. Cell.

[33] Donnelly K.L., Smith C.I., Schwarzenberg S.J., Jessurun J., Boldt M.D., Parks E.J. (2005). Sources of fatty acids stored in liver and secreted via lipoproteins in NAFLD. J. Clin. Investig..

[34] Buzzetti E., Pinzani M., Tsochatzis E.A. (2016). The multiple-hit pathogenesis of non-alcoholic fatty liver disease (NAFLD). Metabolism.

